# Immunopathologic Effects of Prednisolone and Cyclosporine A on Feline Immunodeficiency Virus Replication and Persistence

**DOI:** 10.3390/v11090805

**Published:** 2019-08-30

**Authors:** Craig Miller, Jordan Powers, Esther Musselman, Ryan Mackie, John Elder, Sue VandeWoude

**Affiliations:** 1 Department of Veterinary Pathobiology, Oklahoma State University, Stillwater, OK 74078, USA; 2 Department of Microbiology, Immunology, Pathology, Colorado State University, Fort Collins, CO 80523, USA; 3 Department of Immunology and Microbiology, The Scripps Research Institute, La Jolla, CA 92037, USA

**Keywords:** feline immunodeficiency virus, prednisolone, cyclosporine A, opportunistic disease, therapy, immunopathology, human immunodeficiency virus

## Abstract

Feline immunodeficiency virus (FIV) induces opportunistic disease in chronically infected cats, and both prednisolone and cyclosporine A (CsA) are clinically used to treat complications such as lymphoma and stomatitis. However, the impact of these compounds on FIV infection are still unknown and understanding immunomodulatory effects on FIV replication and persistence is critical to guide safe and effective therapies. To determine the immunologic and virologic effects of prednisolone and CsA during FIV infection, FIV-positive cats were administered immunosuppressive doses of prednisolone (2 mg/kg) or CsA (5 mg/kg). Both prednisolone and CsA induced acute and transient increases in FIV DNA and RNA loads as detected by quantitative PCR. Changes in the proportion of lymphocyte immunophenotypes were also observed between FIV-infected and naïve cats treated with CsA and prednisolone, and both treatments caused acute increases in CD4+ lymphocytes that correlated with increased FIV RNA. CsA and prednisolone also produced alterations in cytokine expression that favored a shift toward a Th2 response. Pre-treatment with CsA slightly enhanced the efficacy of antiretroviral therapy but did not enhance clearance of FIV. Results highlight the potential for drug-induced perturbation of FIV infection and underscore the need for more information regarding immunopathologic consequences of therapeutic agents on concurrent viral infections.

## 1. Introduction

Feline immunodeficiency virus (FIV) is a naturally occurring lentivirus of domestic cats that produces progressive immune depletion resulting in an AIDS-like syndrome [1,2,3,4,5,6,7,8,9,10]. Similar to human immunodeficiency virus (HIV), FIV-infected cats may develop secondary or opportunistic infections as a consequence of viral-induced immune dysfunction, including anterior uveitis, chronic rhinitis, gingivostomatitis and periodontitis, encephalitis and neurologic dysfunction, and lymphoma [10,11,12,13,14,15,16,17,18,19]. While antiretroviral therapy (ART) has demonstrated modest success at controlling FIV infection in vivo, the capacity of ART to alleviate symptoms and control the development of secondary disease syndromes has been limited [9,20,21,22]. Adjunct therapies are frequently used in the clinical environment to ameliorate symptoms of secondary diseases associated with FIV infection, including prednisolone and cyclosporine A (CsA), but the effects of these compounds on FIV infection kinetics are unknown [23,24,25,26,27,28,29,30,31,32]. Thus, there is a critical need for a better understanding of the immunomodulatory effects of these drugs on FIV replication and persistence in order to guide safe and effective therapies during FIV infection.

While prednisolone and CsA differ distinctly in regard to pharmacokinetics and mechanism of action, they produce similar therapeutic effects that are mediated primarily through modulation of interleukin 2 (IL-2) expression [32,33,34,35,36,37,38]. IL-2 is a pro-inflammatory cytokine with many regulatory functions during the development and differentiation of T-lymphocytes [39,40]. Additionally, IL-2 expression plays a central role in the proliferation and activation of effector and memory T-lymphocytes, and both prednisolone and CsA exhibit a similar impact on this important regulatory pathway [39,40]. The primary immunomodulatory effects of prednisolone are achieved through binding of glucocorticoid receptors, followed by nuclear translocation and binding of glucocorticoid response elements (GRE) within target genes [36,37,38]. By this mechanism, prednisolone inhibits gene transcription of inflammatory genes, causing suppression of IL-2 expression that results in decreased T-lymphocyte proliferation [36,37,38]. Alternatively, CsA binds to the intracellular protein receptor cyclophilin and inhibits the phosphatase activity of calcineurin, a critical component of the T-cell activation pathway [32,33,34,35]. By blocking calcineurin activity, CsA inhibits the translocation of the cytosolic component of the nuclear factor of activated T cells (NF-AT), resulting in suppression of IL-2 gene promoter/enhancer function and consequent inhibition of T-lymphocyte proliferation [32,33,34,35]. Thus, although they utilize divergent pharmacokinetic pathways, these compounds produce a similar immunomodulatory effect by decreasing proliferation and activation of T-lymphocytes.

Because FIV predominately targets CD4+ T-lymphocytes for infection in domestic cats [1,2,3,4,6,8], it is possible that immunosuppressive doses of prednisolone and CsA could broadly reduce lymphocyte proliferation and activation in FIV-infected cats, thereby decreasing the number of target cells for FIV infection and as a result, reduce FIV viral and proviral loads in circulation. Alternatively, the immunosuppressive effects of these agents might transiently enhance FIV replication by inhibiting immune responses keeping viral infection at steady-state levels. Thus, to determine the impact of prednisolone and CsA on FIV replication kinetics, chronically infected Specific Pathogen Free (SPF) cats were administered immunosuppressive doses of prednisolone or CsA in a cross-over study design. Following a wash-out period, cats were administered CsA prior to antiretroviral therapy (zidovudine + lamivudine) to assess the effects of immunomodulation on ART efficacy, and to assess whether immunosuppressive therapy would enhance FIV susceptibility to drugs that require viral replication. Alterations in hematological parameters were monitored over time by complete blood count and flow cytometry and correlated with changes in circulating FIV RNA and DNA levels as detected by quantitative PCR (qPCR). Shifts in circulating lymphocyte immunophenotype and virologic parameters were compared with peripheral cytokine levels quantified by microsphere immunoassay (MIA) to assess changes in innate immune function.

Results indicated that both prednisolone and CsA induced acute and transient increases in FIV DNA and RNA levels which correlated with acute increases in lymphocyte subsets during treatment. Moreover, CsA administration prior to the start of ART induced a slight reduction in FIV RNA viral loads during ART treatment, suggesting upregulation of FIV replication by CsA may improve efficacy of nucleoside analog inhibitors. Collectively, these results document effects of both prednisolone and CsA during FIV infection, and highlight the need to assess the long-term immunopathogenic consequences of these immunomodulatory compounds in animals with chronic viral infections.

## 2. Materials and Methods

### 2.1. In vivo Protocols

This study was approved by the Colorado State University (CSU) Institutional Animal Care and Use Committee (IACUC) (Protocol #14-4872A, Molecular Characterization of FIV, 3/27/2014). Colorado State University’s animal care program is licensed by the United States Department of Agriculture (USDA), accredited by Association for Assessment and Accreditation of Laboratory Animal Care (AAALAC) International, and holds an Office of Laboratory Animal Welfare (OLAW) assurance (A3572-01). All animal experiments complied with the National Institutes of Health guide for the care and use of laboratory animals (NIH Publications No. 8023, revised 1978). Prior to experimental procedures, all study animals were anesthetized by intramuscular injection of ketamine (20 mg/kg) and acepromazine (2 mg/kg) to minimize animal suffering and distress. All study animals were monitored daily by animal care personnel for development of clinical signs of FIV infection and observed by clinical veterinarians.

**Figure 1:**
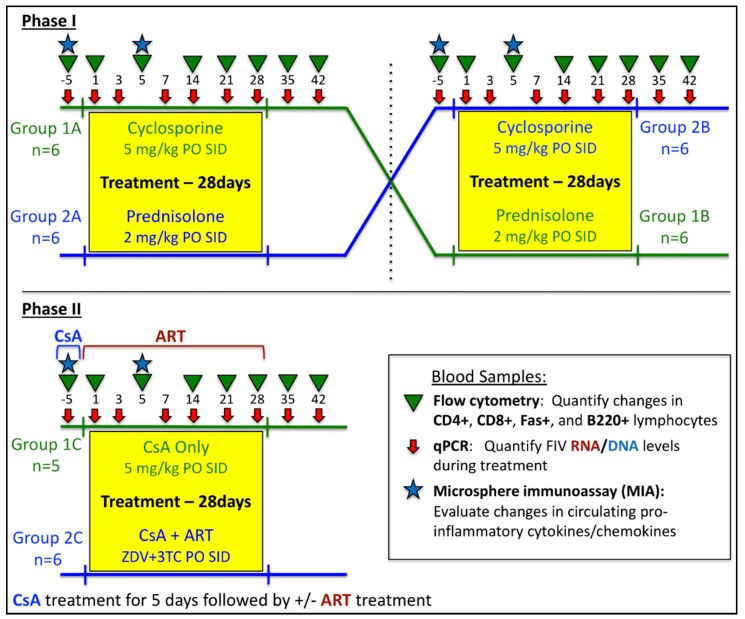
Study design to assess effects of immunosuppressive therapies on feline immunodeficiency virus (FIV) infection. Phase I: Twelve cats were divided into two groups (*n* = 6) and treated with either ATOPICA^®^ for Cats (cyclosporine A, CsA)(5 mg/kg per os (PO) once a day (SID)) or prednisolone (2 mg/kg PO SID) for 28 days. Following a 6-week wash-out period, groups were alternated so that each cat received the opposite treatment for 28 days. Phase II: Following a 6-week wash-out period from Phase I, eleven cats received CsA for 5 days, after which, six cats received antiretroviral therapy (ART + CsA) for 28 days (zidovudine 10 mg/kg + lamivudine 50 mg/kg, PO BID), while the remaining five cats received no further treatment (CsA Only). See Materials and Methods for additional details. ART, antiretroviral therapy.

An outline of the study design is presented in Figure 1. Twelve (12) SPF cats from the Andrea D. Lauerman Specific Pathogen Free Feline Research Colony (Fort Collins, CO, USA) were housed as previously described above in accordance with CSU IACUC-approved protocols and were acclimated to the facility for 2 weeks prior to initiation of the study. As part of an FIV vaccination protocol [41], animals were intravenously inoculated with 75,000 infectious units of FIV_PPR_ (1 mL of a viral stock solution with a TCID_50_ titer of 1 × 10^5.87^) at 8–11 weeks of age. At the onset of this study, all animals were between 43–46 weeks of age and were confirmed to be FIV-positive by quantitative PCR detection of viral RNA [41].

Phase I: FIV-positive cats were divided into 2 groups (*n* = 6, randomized for previous vaccine treatment, gender, and litter) and treated as follows: Group 1A—ATOPICA^®^ for Cats (cyclosporine A oral solution (CsA), Novartis, East Hanover, NJ, USA) at 5 mg/kg PO SID for 28 days; Group 2A—prednisolone 2 mg/kg PO SID for 28 days. Following this treatment protocol, all cats were subjected to a wash-out period of 6 weeks, in which no cats received either treatment. During this wash-out period, blood measurements of drug concentration were evaluated every 2 weeks until no detectable amounts of the previously administered drug were detected in treated cats. Blood samples were collected at days −5, 1, 3, 5, 7, 10, 14, 21, 28, 35, 42 of Phase I for quantitative polymerase chain reaction (qPCR), complete blood counts (CBC), and flow cytometry analysis using previously established protocols [10,42] (Figure 1). Following the 6-week wash-out period, drug treatment protocols for each cat group were alternated so that each cat received treatment as follows: Group 1B—prednisolone 2 mg/kg PO SID × 28 days; Group 2B—ATOPICA^®^ (CsA) at 5 mg/kg PO SID for 28 days. Blood samples were collected as previously described for qPCR, CBC, and flow cytometry analysis (Figure 1). For Phase I, uninfected, age-matched negative control cats (naïve control)(*n* = 12) were included, divided into 2 groups (*n* = 6), and administered either ATOPICA^®^ (CsA, 5 mg/kg PO SID) or prednisolone (2 mg/kg PO SID) for 28 days as previously described.

Phase II: Following a 6-week wash-out period from Phase I, all FIV-infected cats were pre-treated with CsA for 5 days prior to day 0, after which CsA treatment ceased and a subset of cats were then administered ART for 28 days. As outlined in Figure 1, FIV-positive cats were divided into 2 randomized groups and treated as follows: Group 1C—(CsA Only, *n* = 5) CsA at 5 mg/kg PO SID for 5 days prior to day 0; Group 2C—(CsA + ART, *n* = 6) CsA at 5 mg/kg PO SID for 5 days prior to day 0, followed by zidovudine (ZDV) 10 mg/kg + lamivudine (3TC) 50 mg/kg, PO BID for 28 days. Blood samples were collected for qPCR, CBC, and flow cytometry analysis as outlined in Figure 1 [10,42].

### 2.2. Hematologic Analyses

Complete blood counts and serum biochemistry analysis were performed for all blood samples in Phase I and Phase II by the CSU Veterinary Diagnostic Lab (CSU-VDL). Blood was collected from all cats prior to the study (day -5) to establish baseline values, then at each time point indicated in Figure 1. The percentage of cells positive for CD4, CD8, Fas, and B220 surface antigens was determined by incubating 30 µL of EDTA-treated blood from each cat in 96-well round-bottom plates with 0.6 µL of RPE-labeled anti-feline CD4 (Southern Biotech; clone 3-4F4, Birmingham, AL, USA), FITC-labeled anti-feline CD8 (Southern Biotech; clone fCD8), PE/Cy7-labeled anti-feline CD45R/B220 (Biolegend; clone RA3-6B2, San Diego, CA, USA), and APC/Cy7-labeled anti-feline Fas/TNFRSF6 (R&D Systems; clone 431006, Minneapolis, MN, USA) mouse monoclonal antibodies diluted in FACS buffer (5% BSA, 0.1% sodium azide in PBS). Following incubation for 30 min in the dark at room temperature, red blood cells (RBCs) were lysed, and stained cells were fixed using a Beckman Coulter Q-Prep work station with 600 µL of 0.1% Formic Acid, 270 µL of 0.06 M Na_2_CO_3_ anhydrous, 0.25 M NaCl, 0.25 M Na_2_SO_3_, and 90 µL 1% wt/vol paraformaldehyde in 1 × PBS. Flow cytometry was performed on a Coulter Gallios (Beckman Coulter Inc, Brea, CA, USA) and results were analyzed using FlowJo^®^ software (FlowJo, Ashland, OR, USA). The percentage of lymphocytes positive for each marker was evaluated over time and compared to baseline values (day −5) and naïve control data to compare alterations in lymphocyte immunophenotype in response to treatment and in the presence of FIV infection. Immunophenotype cell counts were calculated as previously described [42,43] and compared with CBC and qPCR data to evaluate changes in circulating immunophenotype compared to FIV viral and proviral loads over the course of the study and at individual time points. All CD4, CD8, and CD45R/B220 antibodies were directly labeled by the manufacturer. Anti-Fas antibody was unlabeled but subsequently conjugated to APC/Cy7 using a APC/Cy7^®^ Labeling Kit (Abcam, Cambridge, UK).

### 2.3. Quantification of FIV Viral RNA and Proviral DNA in Blood

Blood samples collected during Phase I and Phase II were analyzed by quantitative polymerase chain reaction (qPCR) analysis to quantify FIV proviral DNA and FIV *gag* RNA at time points illustrated in Figure 1. Plasma was isolated from EDTA-treated whole blood following centrifugation and frozen at −70 °C until processing. Viral RNA was extracted from 140 µL plasma using a QIAamp Viral RNA Mini Kit (Qiagen, Valencia, CA, USA) according to the manufacturer’s instructions. Viral RNA from each sample was converted to cDNA using Superscript II reverse transcriptase (Invitrogen, Carlsbad, CA, USA) in individual reactions with random hexamers (Invitrogen) and then treated with RNase Out (Invitrogen) prior to real-time PCR quantification. Peripheral blood mononuclear cells (PBMC) from all cats were purified on a Histopaque (Sigma, St. Louis, MO, USA) gradient, washed, pelleted, and then frozen at −80 °C. Proviral DNA was extracted from PBMCs using a DNeasy Blood and Tissue Kit (Qiagen, Valencia, CA, USA) prior to real-time PCR quantification.

Real-time PCR reactions were performed on a CFX96™ Real-Time PCR Detection System (Bio-Rad, Hercules, CA, USA) to detect and quantify FIV proviral DNA in PBMCs and FIV *gag* RNA in plasma using previously described FIV-A primers and probes [44], and an iTaq™ Universal Probes Supermix (Bio-Rad, Hercules, CA) containing an antibody-mediated hot-start iTaq DNA polymerase. Copy numbers of viral RNA in plasma was calculated as previously described [42,43], implementing a standard curve generated by diluting FIV-PPR virus stock in naïve cat plasma and analyzed by reverse-transcriptase qPCR as outlined above. To quantify proviral DNA in PBMCs, a real-time PCR standard curve was generated from serial dilutions of feline PBMCs from 1 × 10^3^ to 5 × 10^6^ subjected to real-time PCR for the cellular house-keeping gene, Glyceraldehyde-3-Phosphate Dehydrogenase (GAPDH) as previously described [43,45]. Resulting proviral copy numbers were normalized to copies per 10^6^ cells based on the total amount of DNA present in the reaction (100 ng).

### 2.4. Evaluation of Peripheral Cytokine Expression during Immunomodulatory Therapy

Plasma samples collected at day −5 (pre-treatment) and day 5 (post-treatment) of Phase I (Figure 1) were analyzed by a commercially available MILLIPLEX^®^ MAP Feline Cytokine/Chemokine Magnetic Bead Panel (fluorophore-conjugated microspheres, Millipore, Burlington, MA, USA) per manufacturer’s instructions. Briefly, 25 μL from each triplicate sample was combined into one sample per animal. A quantity of 50 μL of each combined sample was incubated with a composite panel of microspheres coupled with capture antibodies to INFγ, IL-1β, IL-2, IL-8, IL-10, MCP-1,TNFα, Fas, SDF-1, SCF, RANTES, PDGF-BB, KC, IL-18, IL-13, IL-12 (p40), IL-6, IL-4, GM-CSF, and Flt-3 ligand. Following incubation with biotinylated secondary antibodies and streptavidin-conjugated phycoerythrin (PE), soluble cytokine molecules were detected in each sample using a Bio-Plex^®^ 200 detection system. Final analyte concentration was calculated using manufacturer-provided standard curves for each analyte and Bio-Plex Manager™ 5.0 software (Bio-Rad).

### 2.5. Statistical Analysis

All analyses were conducted in the program R v3.0.2 (www.r-project.org) using the “stats” package or using GraphPad Prism 6.0 software (La Jolla, CA, USA). *p*-values ≤ 0.05 were considered significant. Repeated-measures ANOVA with multiple comparisons was used to evaluate differences in viral RNA (copies/mL), proviral DNA (copies/10^6^ PBMC), lymphocyte immunophenotype (percentage), and cytokine concentration (pg/mL) among treatment groups over time and at individual time points. Pearson correlation with linear regression was used to evaluate the association between viral RNA, proviral DNA, and lymphocyte immunophenotype over time. Viral RNA, proviral DNA, and cytokine concentration was log transformed to achieve normality prior to analysis.

## 3. Results

### 3.1. Prednisolone and Cyclosporine A Induce Acute and Transient Increases in Circulating FIV Viral and Proviral Loads

**Figure 2:**
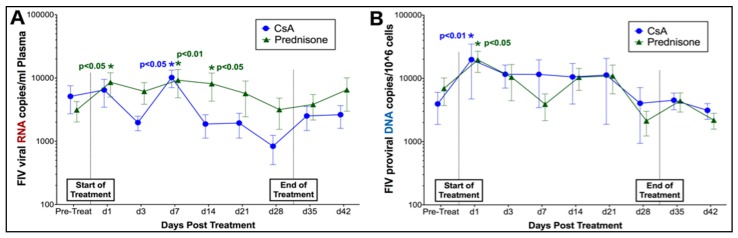
Prednisolone and CsA induce acute and transient increases in FIV RNA and DNA. (**A**) Prednisolone-treated cats (*n* = 12 total; Group 2A + Group 1B) exhibited significantly increased levels of FIV RNA at days 1, 7, and 14 compared to pre-treatment measurements, while CsA-treated cats (*n* = 12 total; Group 1A + Group 2B) had increased FIV RNA at day 7 post-treatment. (**B**) Both prednisolone and CsA-treated cats exhibited significantly increased levels of FIV DNA at 1 day post-treatment.

Phase I. Quantitative PCR analysis of plasma detected FIV RNA in study animals prior to treatment with both prednisolone and CsA, and at all time points during and following treatment (Figure 2). Changes in FIV viral copies per mL of plasma were compared over time to detect changes in RNA viral load during treatment. Although FIV RNA viral loads did not differ over time between treatment groups in this study, significant differences in FIV RNA were detected at individual time points when compared to pre-treatment FIV RNA viral loads. Levels of FIV RNA were significantly elevated in plasma of prednisolone-treated cats at days 1, 7, and 14 during treatment, and were also transiently increased at day 7 of CsA treatment (Figure 2A). Similarly, FIV proviral DNA loads did not differ between treatment groups over time; however, levels of FIV DNA were significantly increased in PBMCs of both prednisolone- and CsA-treated cats at 1 day of treatment (Figure 2B).

**Figure 3:**
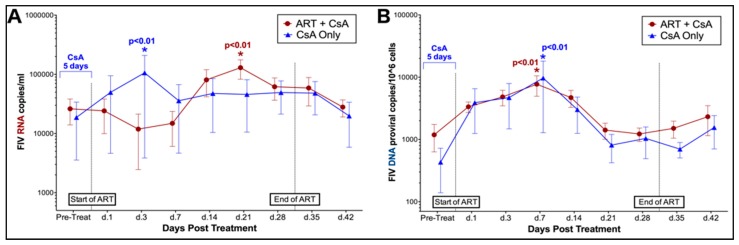
CsA induces recurrent increases in FIV RNA and DNA. (**A**) Cats treated only with CsA (CsA Only) exhibit a transient increase FIV RNA at day 3 post-treatment. FIV-infected cats pre-treated with CsA exhibit a slight decrease in FIV RNA at days 3 and 7 of antiretroviral treatment, but viral loads are significantly increased by day 21. (**B**) Cats treated with CsA alone and CsA + ART exhibit significantly increased levels of FIV DNA at day 7 post-CsA treatment.

Phase II. Based upon findings in Phase I, cats were treated with CsA prior to antiretroviral therapy (ART) to evaluate the potential of CsA to amplify the effects of antiretroviral therapy during an active state of FIV replication. Quantitative PCR was used to evaluate changes in FIV RNA and DNA viral and proviral loads over time in response to therapy. As previously observed in Phase I, cats exhibited a significant but transient increase in FIV RNA in the acute stage of CsA treatment (CsA Only group) (day 3, *p* < 0.01) (Figure 3A). Although CsA + ART cats exhibited a slight decrease in FIV RNA levels at days 3 and 7 of ART, this finding was not sustained and was not statistically significant. Similar to previous results, FIV RNA levels increased after day 7 in cats treated with CsA + ART, resulting in significantly increased viral loads at day 21 (*p* < 0.01) (Figure 3A). Interestingly, acute and transient increases in FIV proviral DNA were observed in cats treated with both CsA Only and CsA + ART at day 7 (*p* < 0.01) (Figure 3B).

### 3.2. FIV-Infected Cats Exhibit Divergent Lymphocyte Immunophenotypes during Cyclosporine A and Prednisolone Treatment

**Figure 4:**
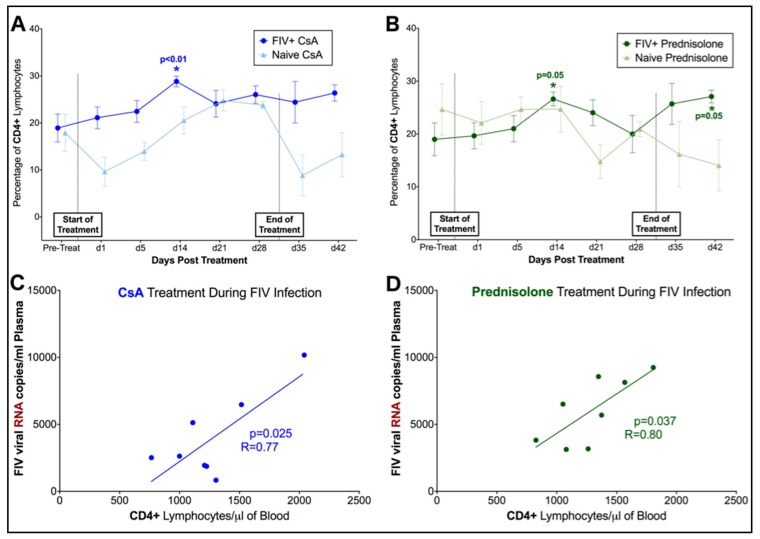
CsA and prednisolone induce a transient increase in CD4+ lymphocytes in FIV-infected cats that correlates with increased FIV viral load. CD4+ lymphocytes are significantly increased in FIV-infected cats at day 14 of CsA treatment (**A**), and at day 14 of prednisolone treatment (**B**). Increased levels of CD4+ lymphocytes correlate with increased levels of FIV RNA in CsA-treated cats (**C**) and prednisolone-treated cats (**D**). See Materials and Methods for additional details.

Phase I. Flow cytometry was used to evaluate changes in the percentage of lymphocytes positive for CD4, CD8, Fas, and B220 surface antigens over time in FIV-infected and naïve cats. Changes in FIV-infected cats were compared to baseline values and data from naïve control cat samples to compare alterations in lymphocyte immunophenotype in response to treatment and in the presence of FIV infection. Overall, the percentage of CD4+ lymphocytes differed significantly over time in FIV-infected cats treated with both CsA (interaction, *p* = 0.05) and prednisolone (interaction, *p* = 0.03) when compared to FIV-negative control cats. Post-hoc analyses indicated that FIV-infected cats exhibited transient increases in CD4+ lymphocytes at 14 days of treatment with both CsA (*p* = 0.004) and prednisolone (*p* = 0.05) when compared to baseline data (pre-treatment) (Figure 4A,B). When compared to FIV viral and proviral loads, a significant positive correlation was observed between numbers of CD4+ lymphocytes and circulating copies of FIV RNA in CSA (*p* = 0.025) and prednisolone-treated cats (*p* = 0.037) (Figure 4C,D). Although the proportion of CD4+ lymphocytes was decreased in naïve control animals at day 1 of CsA treatment and immediately following cessation of treatment (day 35) when compared to baseline data (pre-treatment), changes in this group were not statistically significant, and the percentage of CD4+ lymphocytes did not differ over time during CsA and prednisolone treatment in control animals.

**Figure 5:**
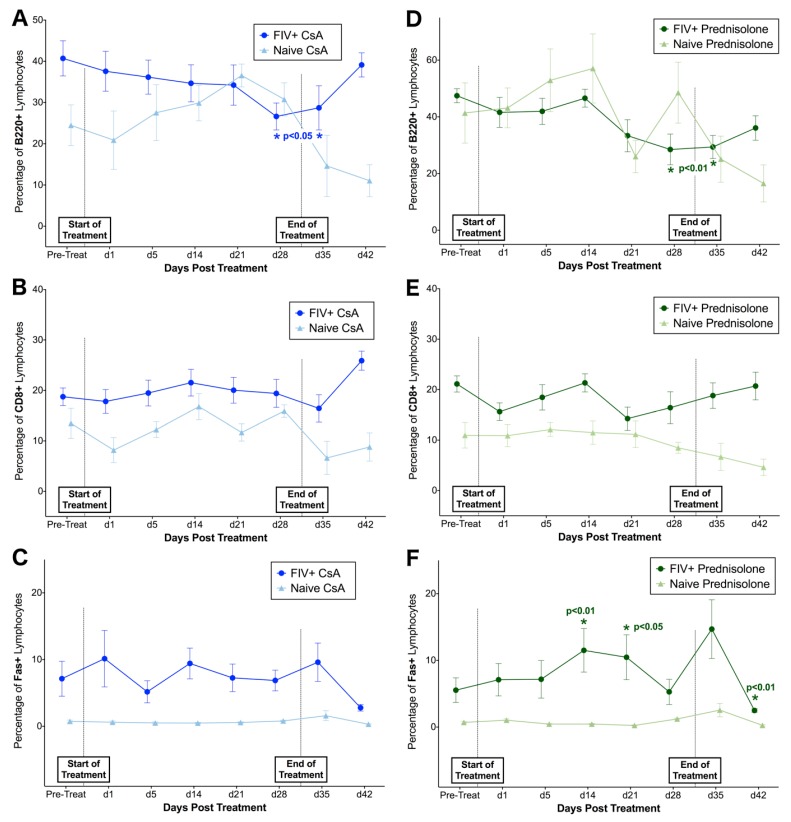
B220+ lymphocytes decrease over time in FIV-infected cats treated with CsA and prednisolone. (**A**) B220+ lymphocytes are significantly decreased in FIV-infected cats after 28 days of treatment with CsA. A reverse trend was observed in naïve animals treated with CsA; however, these changes were not statistically significant when compared to baseline data. (**B**,**C**) No significant differences in CD8+ or Fas+ lymphocytes are observed in FIV-infected cats treated with CsA when compared to baseline levels or naïve controls. (**D**) B220+ lymphocytes are significantly decreased in FIV-infected cats at 28 days of treatment with prednisolone. No significant changes in CD8+ lymphocytes are observed in FIV-infected cats during Phase I (**E**). However, Fas+ lymphocytes are significantly increased at days 14 and 21 of prednisolone treatment in FIV-infected cats (**F**), although this subpopulation of lymphocytes significantly decreases with cessation of treatment (day 42).

When compared to naïve controls, the proportion of B220+ lymphocytes differed significantly over time in FIV-infected cats treated with CsA (interaction, *p* = 0.02) and prednisolone (interaction, *p* = 0.02) (Figure 5A,D), and the proportion of B220+ lymphocytes gradually decreased in FIV-infected cats treated with both compounds. A reverse trend was observed in CsA-treated animals, with an apparent increase in B220+ lymphocytes over time in the naïve control group. However, changes in B220+ lymphocyte percentage were not statistically significant in naïve animals when compared to baseline data. In contrast with changes in CD4+ lymphocytes, FIV-infected cats exhibited significant decreases in B220+ lymphocytes after 28 days of treatment with CsA (*p* = 0.033–0.013) and after 21 days of prednisolone (*p* = 0.034–0.005). No significant differences were detected in CD8+ lymphocytes between FIV-infected cats and naïve controls or in response to CsA or prednisolone treatment (Figure 5B,E), and Fas+ lymphocytes did not differ between FIV-infected and naïve cats treated with CsA (Figure 5C). However, the percentage of Fas+ lymphocytes differed significantly over time in FIV-cats treated with prednisolone (*p* = 0.037) compared to naïve controls, and post-hoc analysis revealed transient increases in Fas+ lymphocytes at 14 days (*p* = 0.004) and 21 days (*p* = 0.016) of prednisolone treatment, followed by a significant spike in Fas+ cells with cessation of treatment (day 35, *p* < 0.0001) (Figure 5F). The percentage of CD8+, B220+, and Fas+ lymphocytes did not differ over time during CsA and prednisolone treatment in naive control animals.

**Figure 6:**
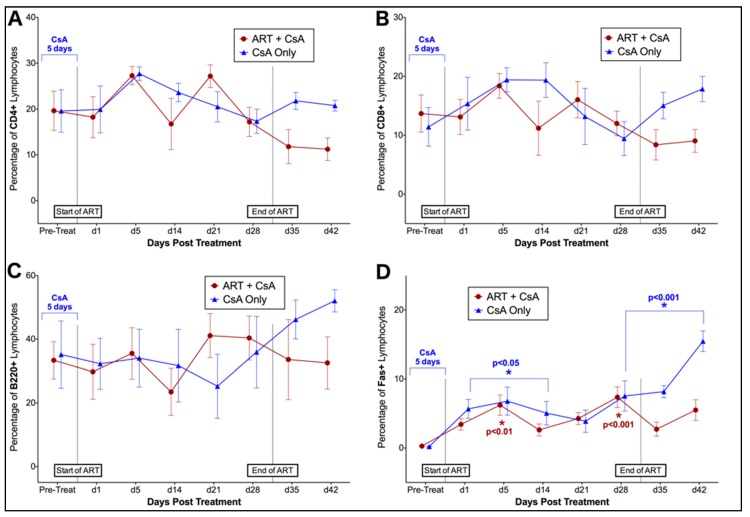
Pre-treatment with CsA prior to ART does not result in significant changes in lymphocyte immunophenotype. No significant differences between (**A**) CD4+, (**B**) CD8+, or (**C**) B220+, are observed following 5 days of CsA Treatment (CsA Only group, *n* = 5; CsA + ART group, *n* = 6) or with 28 days or antiretroviral therapy (CsA + ART group, *n* = 6). The proportion of Fas+ lymphocytes (**D**) increases over time in both CsA only- and CsA + ART-treated animals when compared to baseline (pre-treatment) data.

Phase II. Flow cytometry was used to evaluate changes in the percentage of lymphocytes positive for CD4, CD8, Fas, and B220 surface antigens over time in FIV-infected cats in response to treatment with CsA with or without antiretroviral therapy (CsA only, CsA + ART). Over the course of Phase II, no significant differences in CD4+, CD8+, or B220+ lymphocytes were observed in response to 5 days of CsA treatment, or in response to 28 days of ART (Figure 6A–C). However, the proportion of Fas+ lymphocytes changed significantly over time in both CsA only and CsA + ART treated animals (*p* < 0.0001) (Figure 6D). Cats treated with CsA Only exhibited significantly increased Fas+ cells at day 1, 5, 14, 28, 35, 42 post-treatment, whereas CsA + ART treated cats exhibited increased Fas+ cells at day 5 and 28 (when compared to baseline data).

### 3.3. Peripheral Cytokine Expression is Altered by Prednisolone and CsA during FIV Infection

**Figure 7:**
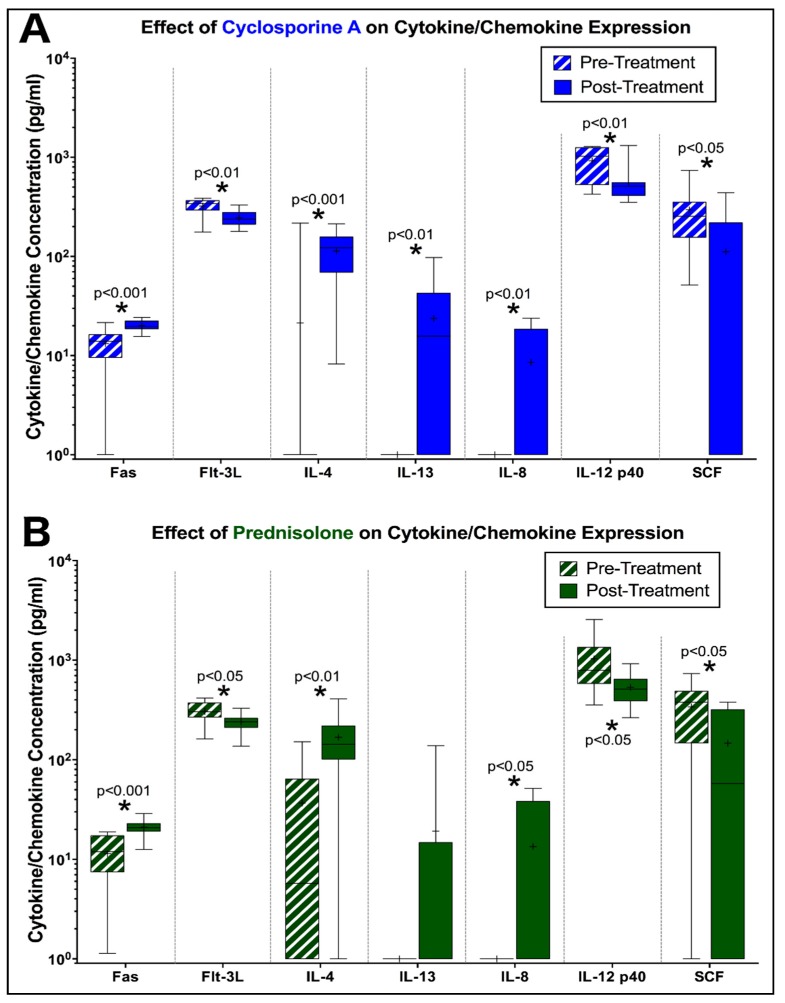
FIV-infected cats treated with CsA and prednisolone exhibit alterations in circulating cytokines. (**A**) FIV-infected cats exhibit increased levels of Fas, IL-4, IL-13, and IL-8 at 5 days of CsA treatment, while levels of Flt-3L, IL-12 p40, and SCF were decreased compared to pre-treatment levels. (**B**) Prednisolone-treated cats with FIV exhibit similar changes in cytokine expression (increased Fas, IL-4, IL-8 and decreased Flt-3L, IL-12 p40, SCF) at 5 days of treatment. Box and whisker plot, “+” indicates the mean for each data set.

Circulating cytokine and chemokines were quantified in plasma by microsphere immunoassay (MIA) at day -5 (pre-treatment) and day 5 (post-treatment) to detect changes in cytokine expression in FIV-infected cats treated with prednisolone or cyclosporine A. Overall, FIV-infected cats treated with CsA exhibited increased levels of circulating Fas (*p* < 0.001), IL-4 (*p* < 0.001), IL13 (*p* < 0.01), and IL-8 (*p* < 0.01) compared to baseline levels, while levels of Flt-3L (*p* < 0.01), IL-12 p40 (*p* < 0.01), and SCF (*p* < 0.05) were significantly decreased (Figure 7A). Similar findings were observed in FIV-infected cats treated with prednisolone, which exhibited increased levels of circulating Fas (*p* < 0.001), Il-4 (*p* < 0.01), and IL-8 (*p* < 0.05) compared to baseline data, while levels of Flt-3L (*p* < 0.05), IL-12 p40 (*p* < 0.05), and SCF (*p* < 0.05) were comparatively decreased (Figure 7B).

## 4. Discussion

The results of this study represent previously undocumented effects of prednisolone and cyclosporine A during chronic FIV infection and highlight important complications to consider when using immunosuppressive therapy to treat opportunistic disease in FIV-infected cats. Since prednisolone and CsA exhibit well-documented immunosuppressive effects via inhibition of IL-2-mediated T-lymphocyte proliferation and activation [32,33,34,36,37], we hypothesized that treatment with these immunomodulatory agents in FIV-infected cats could result in reduction of circulating lymphocytes necessary for FIV-persistence and reduce the ability of FIV to replicate. However, immunosuppressive doses of CsA and prednisolone in FIV-infected cats transiently exacerbated FIV replication rates in conjunction with acute increases in the proportion of CD4+ lymphocytes and increased proviral loads. Although the mechanisms driving these changes are not yet apparent, this evidence suggests that direct interplay between drug-induced immunosuppression and shifts in circulating lymphocyte populations may result in transient increases in FIV replication, which may then result in higher levels of proviral DNA. Prednisolone has not previously been documented to induce such changes in FIV-infected cats, but CsA has been shown to cause a transient increase in plasma viral loads at 4 weeks (28 days) of treatment in a cohort of FIV-infected cats [46]. This is consistent with the literature documenting similar increases in viral loads and drug-induced shifts in CD4+ lymphocyte populations in HIV patients treated with both prednisolone and CsA [47,48,49], suggesting a causative relationship that provides latent virus with the means to reinitiate infection of naïve T-cells. This direct relationship suggests that both CsA and prednisolone may either prevent CD4+ lymphocyte depletion, enhance maturation of precursors to the CD4+ phenotype, or cause release of CD+ lymphocytes into circulation; thus, resulting in enhanced viral propagation.

In accordance with these findings, we investigated the potential for an innovative immunomodulatory approach using CsA to compliment antiretroviral therapy. We first selected two nucleoside analogue reverse transcriptase inhibitors (NARTIs), zidovudine and lamivudine, which have been previously shown to be effective in vivo at reducing FIV viral loads in chronically infected cats [21]. Because NARTIs exert their primary therapeutic effect during active viral replication through competitive inhibition and reduction in the activity of reverse transcriptase [50,51,52], we theorized that CsA-mediated induction of an enhanced replicative state might improve the efficacy of antiretroviral therapy. We pre-treated all FIV-infected cats with CsA prior to day 0, after which CsA treatment ceased and a subset of cats were then administered ART for 28 days. As previously observed in Phase I, cats that received CsA treatment only (no ART at day 0) exhibited a similar transient increase in FIV RNA viral load, confirming that CsA treatment induces enhancement of FIV replication in infected cats. Interestingly, a slight reduction in viral load was observed in FIV-infected cats receiving CsA then ART, although this change was not sustained and not statistically significant. It is possible that a more prolonged course of CsA treatment may have been more effective. Furthermore, because ART diminished viral replication in CsA + ART-treated cats compared to cats that received CsA Only, it is likely that the CsA-mediated increase in viral load was due to new viral replication/viral activation. The subsequent rise in FIV RNA levels by day 21 of ART may likely reflect the withdrawal of CsA from the treatment protocol, which effectively terminated the activated state of viral replication that was conducive to improving ART efficacy. An obvious limitation of this study is the absence of an “ART Only” control group, in which cats were administered ART without a CsA pre-treatment. Although this was not possible to achieve in the current study due to animal constraints, the inclusion of this control group is necessary to completely determine the effect of the CsA pre-treatment on ART efficacy. Future studies will focus on the collateral evaluation of ART efficacy in the presence and absence of CsA pre-treatment, and will assess whether prolonged combinational therapy with CsA can sustain its perceived antiviral effect.

Appreciable changes in lymphoid immunophenotype also extended to B220+ lymphocytes, which decreased significantly over time during the course of treatment with both CsA and prednisolone. B220 is a heavily glycosylated isoform of CD45R, and a surface antigen expressed in immature and mature naïve B-cells, as well as other lymphocyte subsets such as activated T-cells and dendritic cells [53,54]. It is well documented that at high doses, prednisolone broadly decreases both T and B lymphocytes, but at lower doses, prednisolone produces selective depletion of B lymphocytes [55,56]. It is possible that although we selected a higher, immunosuppressive dose of prednisolone, the dose may have not been high enough to suppress both T and B lymphocytes in our FIV-infected cohort, thus accounting for the selective depression of B220+ cells and not CD4+ T cells. In contrast, cyclosporine A is known to be primarily selective for T lymphocytes, producing less inhibitory effects on their B lymphocyte counterparts [57,58,59]. However, certain subsets of B lymphocytes, such as those responding to thymus independent (TI) antigens, have been shown to be sensitive to CsA treatment, while primed (or activated) T lymphocytes are resistant [59]. Previous studies have shown that FIV is capable of directly activating T lymphocytes, either by primary virus infection or chronic antigenic stimulation [60,61,62], yet does not affect the function of B cells recognizing TI antigens [8,63]. In this present study, the predisposing condition of FIV-infection may, therefore, account for the decreased efficacy of CsA in this study to reduce CD4+ lymphocytes in circulation and provide cause for the selective depletion of CsA-sensitive subpopulation of B lymphocytes.

Prednisolone and CsA are both well-known inducers of apoptosis in lymphocytes [64,65,66,67]. Although Fas cell surface expression (a marker of apoptosis in lymphocytes) was not significantly elevated in in CsA-treated cats in Phase I, it was increased in prednisolone-treated cats in Phase I and also in all CsA-treated cats in Phase II (CsA Only and CsA + ART). Glucocorticoid- and CsA-induced apoptosis occurs primarily via caspase-dependent activation, and predominately occurs independent of the FasL/Fas system [38,65,67]. Fas-induced apoptosis may occur in viral-infected cells via binding of FasL on CD8+ T cells, and may be upregulated in this study as a result of increased infection of CD4+ lymphocytes in prednisolone- and CsA-treated animals [68]. Microsphere immunoassay also detected significant increases in soluble, circulating Fas in FIV-infected cats treated with both CsA and prednisolone in Phase I. Increased levels of apoptosis in CD4+ and CD19+ lymphocytes have been well-described during HIV infection, and have been correlated with viral RNA levels in plasma and a reduction in these important lymphocyte subsets [69,70]. Interestingly, such a correlation was not observed in this study, however, CD4+ lymphocytes were observed to increase in response to CsA and prednisolone treatment, and may have counterbalanced any viral-induced decrease in lymphocyte numbers in the face of continued CD8-mediated apoptosis.

Important changes in cytokine expression during this study included increased Interleukin 4 (IL-4) in both treatment groups and increased IL-13 in CsA-treated cats infected with FIV. IL-4 is produced predominately by T helper 2 (Th2) cells, and confers anti-inflammatory effects on Th1 cells, macrophages, and interferon gamma (IFNγ) production, as well as plasma cell differentiation, IgE class switching, and differentiation of antigen-stimulated naive T cells into the Th2 subset [71,72,73,74]. IL-13 exhibits similar effects on the inhibition of inflammatory cytokines and is functionally related to IL-4 [75]. Increased levels of these anti-inflammatory cytokines have been previously associated with HIV infection [76,77,78], indicating a shift to a predominant Th2 subtype during infection, but these effects have not been well studied in cats with FIV. Furthermore, prior studies have demonstrated that both prednisolone and CsA treatment are associated with decreases in IL-4 expression, yet in this study, circulating IL-4 concentrations increased in response to treatment [79,80,81,82]. The cause for such a shift in cytokine expression due to CsA or prednisolone treatment is not apparent but may be due to underlying viral infection. If FIV infection produces a shift to a Th2 response as observed in HIV, it is possible that the effect of these compounds to increase CD4+ lymphocytes may have stimulated differentiation of these cells along the Th2 pathway, thus increasing expression of these associated cytokines.

FIV-infected cats treated with both prednisolone and CsA also exhibited increased levels of circulating IL-8 when compared to baseline levels (pre-treatment). IL-8 is pro-inflammatory cytokine that is primarily involved in neutrophil chemotaxis, but also exhibits chemotactic activity against T cells and basophils [83]. Studies in HIV have demonstrated significant increases in circulating levels of IL-8 in plasma of infected patients and have shown that IL-8 plays a significant role in stimulating HIV replication [84,85,86]. IL-8 may exhibit similar kinetics during FIV-infection, and the increased rate of FIV replication during prednisolone and CsA treatment may, therefore, account for the corresponding increase in IL-8 expression during immunomodulatory treatment.

In contrast, FIV-infected cats treated with both prednisolone and CsA exhibited a significant decrease in the expression of several pro-inflammatory cytokines, such as IL-12 p40, Flt-3L, and SCF. IL-12 p40 is secreted by active macrophages and is critical in the production of IFNγ and the induction of Th1 cells [87]. Prednisolone and cyclosporine may directly suppress IL-12 p40 expression and likely account for the decreased levels in this study [88,89,90]; however, previous studies have also shown that HIV-infected patients have impaired IL-12 p40 secretion, and the contribution of FIV infection and replication may likewise play a role in this study as well [91]. Flt-3L and SCF play an important role in hematopoiesis and function by increasing lymphocyte proliferation by activating hematopoietic progenitors [92,93,94,95]. Decreased Flt-3L and SCF has been well-documented during prednisolone and CsA treatment, which most likely account for decreased levels of these cytokines, yet the inhibition of these inducers of cell proliferation was not sufficient to cause a decrease in CD4+ lymphocytes in FIV-infected cats [96,97,98]. While the exact mechanism is not clear in this present study, it is likely that CD4+ lymphocyte increases occurred independently of these stimulatory cytokines during treatment with these immunomodulatory agents in FIV-infected cats.

In summary, the results presented herein provide new insight into the use of commonly prescribed immunomodulatory agents during FIV infection. While pre-treatment with CsA may have the potential to reduce viral loads when combined with nucleoside analog reverse transcriptase inhibitors through induction of an active state of viral replication conducive to competitive inhibition, the augmentative effects that prednisolone and CsA alone impart on viral replication kinetics may necessitate caution in their clinical application in FIV-infected cats. Overall, these results underscore the need for more information regarding immunopathologic consequences of therapeutic agents on concurrent viral infections.
